# The immediate effects of private equity acquisition of urology practices on the management of newly diagnosed prostate cancer

**DOI:** 10.1002/cam4.6788

**Published:** 2023-12-15

**Authors:** Kassem S. Faraj, Samuel R. Kaufman, Lindsey A. Herrel, Avinash Maganty, Mary Oerline, Megan E. V. Caram, Vahakn B. Shahinian, Brent K. Hollenbeck

**Affiliations:** ^1^ Dow Division of Health Services Research, Department of Urology University of Michigan Ann Arbor Michigan USA; ^2^ VA Health Services Research & Development, Center for Clinical Management Research, VA Ann Arbor Healthcare System Ann Arbor Michigan USA; ^3^ Division of Hematology/Oncology, Department of Internal Medicine University of Michigan Ann Arbor Michigan USA; ^4^ Division of Nephrology, Department of Internal Medicine University of Michigan Ann Arbor Michigan USA; ^5^ Department of Urology Massachusetts General Hospital Boston Massachusetts USA

**Keywords:** cancer, financial incentives, private equity, prostate cancer

## Abstract

**Introduction:**

Some worry that physician practices acquired by private equity may increase the use of services to maximize revenue. We assessed the effects of private equity acquisition on spending, use of treatment, and diagnostic testing in men with prostate cancer.

**Methods:**

We used a 20% sample of national Medicare claims to perform a retrospective cohort study of men with prostate cancer diagnosed from 2014 through 2019. The primary outcome was prostate cancer spending in the first 12 months after diagnosis. Secondary outcomes included the use of treatment and a composite measure of diagnostic testing (e.g., imaging, genomics) in the first 12 months after diagnosis. Multilevel modeling was used to adjust for differences in patient and market characteristics. The effect of practice acquisition on each outcome was assessed using a difference‐in‐differences design.

**Results:**

There were 409 and 4021 men with prostate cancer managed by urologists in acquired and nonacquired practices, respectively. After acquisition, prostate cancer spending was comparable between acquired and nonacquired practices (difference‐in‐differences estimate $1182, *p* = 0.36). Acquisition did not affect the use of treatment (difference‐in‐differences estimate 3.7%, *p* = 0.30) or the use of diagnostic testing in men who were treated (difference‐in‐differences −5.5%, *p* = 0.12) and those managed conservatively (difference‐in‐differences −2.0%, *p* = 0.82).

**Conclusions:**

In the year following acquisition of urology practices, private equity did not increase prostate cancer spending, the use of treatment or diagnostic testing in men with prostate cancer. Future work should evaluate the effects of private equity acquisition on practice patterns and quality over a longer time horizon.

## 1 INTRODUCTION

As evidenced by epidemiological trends in incidence and mortality,^1^ many prostate cancers are slow‐growing and death from other causes is common. Nonetheless, most men undergo treatment.^2^, ^3^ Because the benefits of treatment are less clear in the setting of medical uncertainty (e.g., competing risks vs. disease biology), management decisions in this context are subject to the discretion of the urologist and thus may be susceptible to nonclinical factors. For example, incentives afforded through practicing ownership of radiation vaults, whereby owners collect additional facility revenue, have been associated with increased use of treatment for prostate cancer in unhealthy men, who are least likely to benefit.^4^ Similarly, urologist practice context is strongly associated with management patterns,^4^, ^5^ though there has been considerable recent reorganization through both vertical and horizontal integration and growing involvement of private equity.^6^, ^7^

The involvement of for‐profit private equity firms in the acquisition of urology practices may have implications for men with prostate cancer.^6^ Private equity firms, which are financed by investors and debt, acquire practices that have potential for growth and aim to increase their value through a variety of strategies, such as reducing labor costs, developing new, profitable service lines, and expanding market share. The ultimate goal of these firms, regardless of the industry, is to subsequently sell the acquired entity within 3–7 years for a profit.^8^ The short time horizon is based on the limited lifespan of the fund that is financed partially by investors, who expect returns on investment at fund closure. In the healthcare setting, the time‐sensitive need to grow practice value to make a profit could affect physician behavior in a manner that manifests as changes in practice patterns. Proponents of private equity involvement suggest that firms may improve quality of care through expansion of new services to patients, which affords convenience and potentially improves adherence to physician recommendations.^9^, ^10^, ^11^ However, others worry that the firms may, at least indirectly, encourage utilization and spending.^12^, ^13^, ^14^

We evaluated the immediate effects of private equity acquisition of urology practices on prostate cancer spending, treatment, and use of diagnostic testing. Findings will inform policymakers, urologists, and referring physicians on the implications of private equity involvement for specialty care, which is an important focus for ongoing investment.^8^

## 2 METHODS

We performed a retrospective cohort study using a 20% national sample of fee‐for‐service Medicare beneficiaries diagnosed with prostate cancer between 2014 and 2019. Follow‐up data were available through December 31, 2020. Men with newly diagnosed prostate cancer were identified using an established algorithm validated against cancer registry data.^15^ We included men aged 66 years and older with entitlement to Medicare Parts A and B to allow for assessment of competing health risks in the year before diagnosis. Those participating in managed care plans were excluded to minimize ascertainment bias. Men were assigned to their primary urologist using established methods that reflect the plurality of interactions for the 12‐month period surrounding the diagnosis.^4^, ^16^ Urologists were linked to their practice using data from the Medicare Data on Provider Practice and Specialty file.^17^ Information regarding urology practice acquisition by private equity was obtained using acquisition reports by Irving Levin Associates and complemented with manual internet searches to confirm acquisitions.

The exposure for our analysis was the acquisition of a urology practice by private equity. The comparison group (nonacquired) included patients in practices that were not acquired by private equity. All acquired practices were organized as single‐specialty groups with three or more urologists. Thus, the nonacquired group was limited to patients who were managed by urologists in single‐specialty groups of three or more urologists. Urologists in the nonacquired group were randomly matched to the acquired group proportionately based on the year of acquisition. For example, of the urologists with acquired practices, 28% had their practice acquired in 2016. Therefore, 28% of urologists whose practices were not acquired were randomly matched as the nonacquired group for that year. The final cohort consisted of physicians who had data present in the year before and after the year of acquisition or matched year (nonacquired). Cohort selection is illustrated in Figure S1.

### 2.1 Outcomes

The primary outcome was annual prprostate cancer spending in the 12 months after diagnosis per newly diagnosed man with prostate cancer. This was adjusted for inflation to 2020 dollars using the Consumer Price Index. This measure, which is price standardized, reflects global utilization of care associated with an International Classification of Diseases (versions 9 and 10) code for prostate cancer. Secondary outcomes included treatment for prostate cancer (i.e., surgery, any form of radiation, or cryotherapy) and a composite measure of diagnostic testing use (i.e., multiparametric MRI, tissue‐based genomics, CT scan, bone scan, prostate biopsy) in men who were and were not treated. All outcomes were assessed for the 12‐month period after the date of diagnosis.

Because physician behavior is most responsive to nonclinical factors when the benefits of treatment are the least clear, we performed a subgroup analysis focusing on men with >75% non‐cancer mortality risk within 10 years of diagnosis.^4^, ^18^ Based on recent clinical trial data^19^ and professional guidelines,^20^, ^21^ these men are the least likely to benefit from treatment due to competing risk of death.

### 2.2 Statistical analysis

We compared patient characteristics according to whether their primary urologist was in a practice acquired by private equity with Pearson's chi squared test. To assess the effect of private equity acquisition on prostate cancer spending, we fit a multilevel mixed linear model with a negative binomial distribution, log link function, and robust standard errors. The remaining outcomes were assessed using a multilevel logistic model with robust standard errors. All models incorporated a random intercept at the urologist level to account for clustering of patients within individual urologists. Models were adjusted for patient age, race, socioeconomic class measured at the five‐digit zip code level,^22^ comorbidity,^23^ rural residence, calendar year, practice size, and market characteristics (supply of urologists, radiation oncologists, hospital beds, and Medicare‐managed care penetration).

Using the adjusted models, we performed a difference‐in‐differences analysis to quantify the change in spending from the pre‐ to the post‐acquisition year or matched year (nonacquired) by assessing an interaction term between private equity acquisition and time (before or after acquisition). The estimate from this interaction term represents the difference in the change in spending in men managed by urologists whose practices were acquired relative to those managed by urologists whose practices were not acquired. We confirmed that trends in each outcome were parallel for private equity acquired and nonacquired practices prior to the acquisition period (Figure S2). A similar approach was deployed to estimate the effects of private equity on the use of treatment, spending in men who were treated, and the use of diagnostic testing. We then used a similar approach to assess prostate cancer spending and treatment in men with >75% non‐cancer mortality risk within 10 years of diagnosis.

### 2.3 Sensitivity analyses

The difference‐in‐differences analysis relies on an adequate comparison group that is comparable to the exposure group, except for the intervention that is being studied.^24^ To ensure that the observed changes in outcomes are due to the intervention rather than other factors, we first performed a sensitivity analysis assessing annual prostate cancer spending and the use of treatment using a comparison group that consisted solely of patients who were managed by urologists whose practices were acquired by private equity after the study period and not originally included in the exposure group. This approach aimed to minimize the possibility that changes attributed to the acquisition were due to an inadequate comparison group. Since the practices that are eventually acquired represent desirable acquisition potential, this would help strengthen the validity of findings from our broader analysis. We also performed an additional sensitivity analysis that only included urologists who managed patients in all years of the study.

All analyses were carried out using Stata 17 (College Station, TX). Adjusted probabilities were derived using the margins command in Stata. All tests were two‐sided with probability of Type 1 error (*α*) set at 0.05. The study protocol was judged to be exempt by the institutional review board at our institution.

## 3 RESULTS

Between 2014 and 2019, there were 26,106 men diagnosed with prostate cancer managed by single specialty urology practices with at least 1 year of follow‐up. In the year before acquisition, there were 409 patients managed at practices that were acquired by private equity, and 4021 managed at nonacquired practices. Patient demographics and urology practice market characteristics varied by private equity acquisition at baseline; however, these differences were modest in magnitude (Table 1). Notably, patients who were managed at acquired practices had higher socioeconomic status (47% vs. 35%, *p* < 0.001), were more often black (13% vs. 9%, *p* = 0.02), resided in urban areas (96% vs. 84%, *p* < 0.001) with a high concentration of urologists (46% vs. 37%, *p* < 0.001) and low Medicare advantage penetration (41% vs. 33%, *p* = 0.004), and were managed at large practices (90% vs. 52%, *p* < 0.001). Prior to acquisition, prostate cancer spending was higher for patients managed by urologists whose practices were acquired compared to patients managed by urologists whose practices were not acquired ($18,660 vs. $17,145, *p* = 0.045).

**TABLE 1:** Characteristics of patients in practices acquired by private equity compared to nonacquired practices.

	Acquired			Nonacquired		
	Before	After	*p‐*value	Before	After	*p*‐value
Number of patients	409	357		4021	3243	
Mean age (SD)	73 (5)	73 (5)	0.14	73 (5)	73 (5)	0.30
Race (%)			0.45			0.091
White	327 (80)	272 (76)		3398 (85)	2779 (86)	
Black	52 (13)	54 (15)		351 (9)	241 (7)	
Other	30 (7)	31 (9)		272 (7)	224 (7)	
Comorbidity (%)			0.76			0.074
0	224 (55)	193 (54)		2267 (56)	1834 (57)	
1	82 (20)	64 (18)		790 (20)	584 (18)	
2	53 (13)	54 (15)		522 (13)	414 (13)	
3	50 (12)	46 (13)		442 (11)	411 (13)	
Urologist per 100,000 (%)			0.031			0.35
Low (≤34)	89 (21)	83 (23)		1255 (31)	1003 (31)	
Intermediate	137 (33)	89 (25)		1291 (32)	1094 (34)	
High (≥71)	183 (46)	185 (52)		1475 (37)	1146 (35)	
Radiation oncologist per 100,000 (%)			0.043			0.092
Low (≤13)	94 (23)	69 (19)		1280 (32)	993 (31)	
Intermediate	148 (36)	110 (31)		1191 (30)	1040 (32)	
High (≥29)	167 (41)	178 (50)		1550 (38)	1210 (37)	
Hospital bed per 100,000 (%)			0.090			0.48
Low (≤3340)	109 (27)	102 (29)		1204 (30)	1011 (31)	
Intermediate	218 (53)	164 (46)		1356 (34)	1091 (34)	
High (≥6342)	82 (20)	91 (25)		1461 (36)	1141 (35)	
Medicare advantage penetration			0.30			0.41
Low (≤13.5%)	166 (41)	165 (46)		1308 (33)	1030 (32)	
Intermediate	122 (30)	98 (27)		1361 (34)	1074 (33)	
High (≥25.0%)	121 (29)	94 (26)		1352 (34)	1139 (35)	
Socioeconomic status (%)			0.62			0.094
Low	88 (22)	68 (19)		1177 (29)	898 (28)	
Medium	127 (31)	120 (34)		1436 (36)	1132 (35)	
High	194 (47)	169 (47)		1408 (35)	1213 (37)	
Practice size						
Large	367 (90)	329 (92)	0.25	2093 (52)	1870 (58)	<0.001
Urban residence (%)	394 (96)	342 (96)	0.70	3359 (84)	2736 (85)	0.27

**FIGURE 1:**
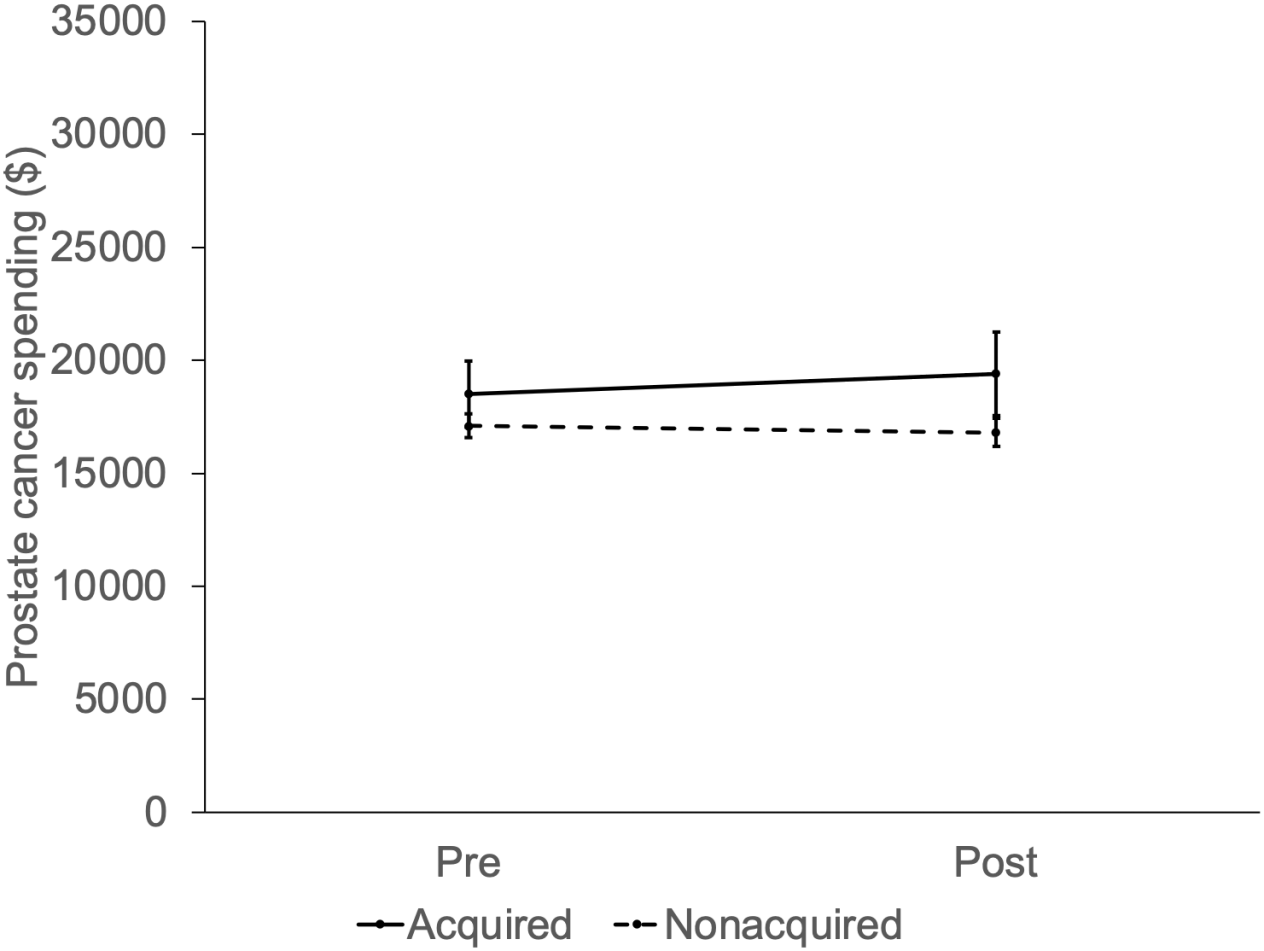
Adjusted prostate cancer spending by practice equity acquisition (not acquired vs. acquired) over time, relative to the acquisition period (pre, post). Private equity did not affect prostate cancer spending in men managed by urologists in practices acquired compared to practices not acquired (difference‐in‐differences $1182, *p* = 0.36).

**FIGURE 2:**
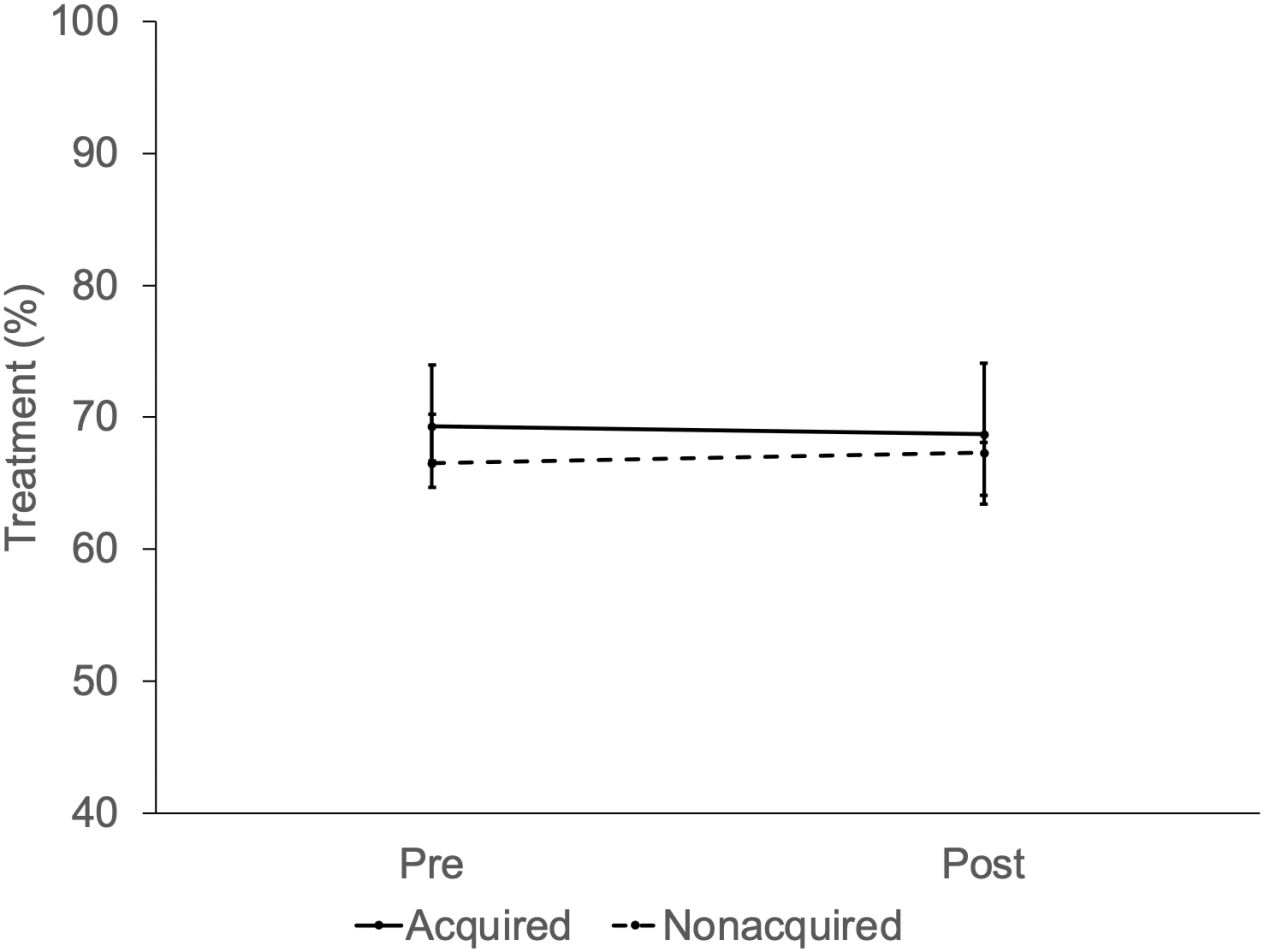
Adjusted percentage of men with prostate cancer treated within 1 year of diagnosis by practice equity acquisition (not acquired vs. acquired) over time, relative to the acquisition period (pre, post). Private equity did not affect treatment in men managed by urologists in practices acquired compared to practices not acquired (difference‐in‐differences ‐3.7%, *p* = 0.31).

**FIGURE 3:**
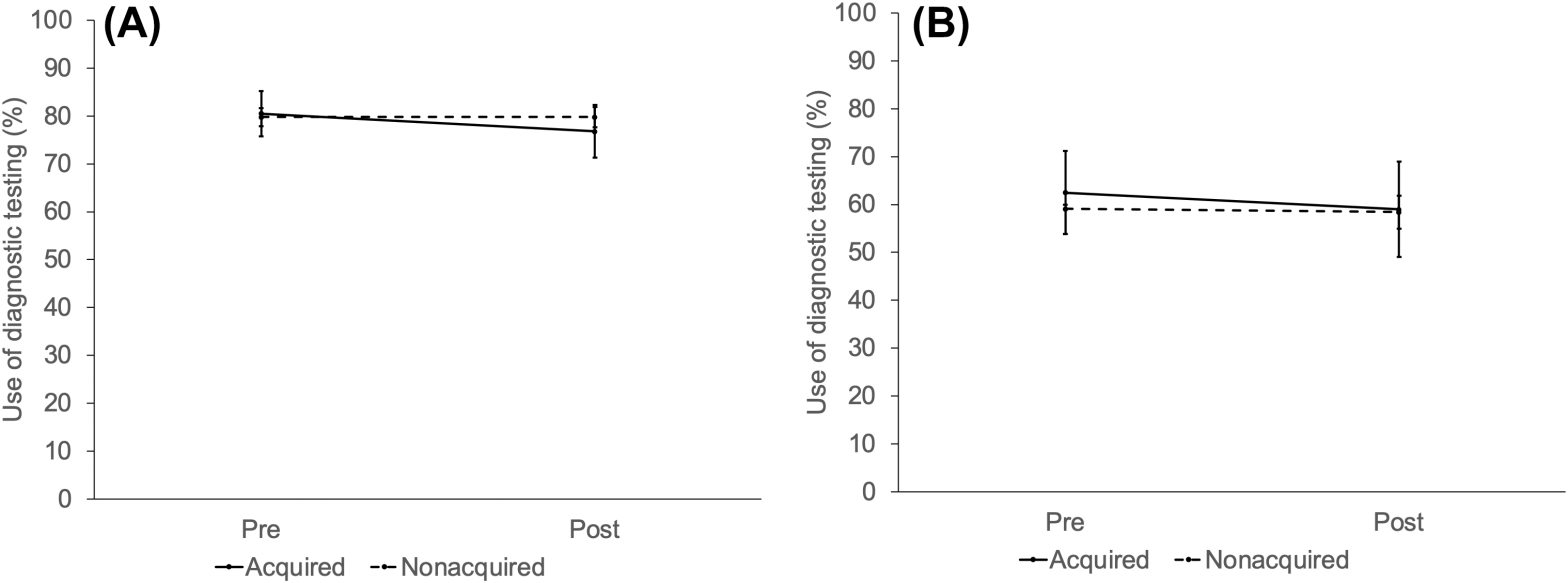
(A) Adjusted percentage of men treated for prostate cancer undergoing diagnostic testing within 1 year of diagnosis by practice equity acquisition (not acquired vs. acquired) over time, relative to the acquisition period (pre, post). Private equity did not affect the use of diagnostic testing in those who were treated (difference‐in‐differences −3.7%, *p* = 0.30). (B) Adjusted percentage of untreated men with prostate cancer undergoing diagnostic testing within 1 year of diagnosis by practice equity acquisition (not acquired vs. acquired) over time, relative to the acquisition period (pre, post). Acquisition did not affect the use of diagnostic testing in untreated men (difference‐in‐differences −5.0%, *p* = 0.82) treated.

After adjusting for patient and market characteristics, private equity acquisition did not significantly increase overall prostate cancer spending (Figure 1; difference‐in‐differences $1182, *p* = 0.36). Private equity acquisition did not affect the use of treatment (Figure 2; difference‐in‐differences −3.7%, *p* = 0.31) or the use of diagnostic testing (Figure 3) in those who were treated (difference‐in‐differences −3.7%, *p* = 0.30) and those who were managed conservatively (difference‐in‐differences −5.0%, *p* = 0.82). In men with >75% non‐cancer mortality risk, private equity acquisition did not result in higher spending (difference‐in‐differences −$1557, *p* = 0.71) or treatment (difference‐in‐differences 8.0%, *p* = 0.71).

The results of the sensitivity analyses were consistent with the findings from the main model specification strategy. First, when using a nonacquired group consisting of urologists whose practices were acquired after the study period, we found that prostate cancer spending was no different by urologists whose practices were acquired compared to patients managed by urologists whose practices were not acquired ($18,660 vs. $18,677, *p* = 0.99). Further, acquisition did not lead to an increase in overall prostate cancer spending (difference‐in‐differences $2024, *p* = 0.18) or use of treatment (difference‐in‐differences −2.1%, *p* = 0.64). Second, when assessing outcomes of patients whose urologists were available for all years of the analysis, we found that acquisition did not lead to an increase in overall prostate cancer spending (difference‐in‐differences $1802, *p* = 0.46) or use of treatment (difference‐in‐differences −1.9%, *p* = 0.64).

## 4 DISCUSSION

Private equity acquisition of urology practices does not increase overall price standardized prostate cancer spending in the year after acquisition. Additionally, acquisition did not increase the use of treatment or diagnostic testing. There was also no change in spending or treatment when assessing a subgroup of men with >75% non‐cancer mortality risk within 10 years of diagnosis. The results of this study suggest that private equity acquisition does not affect the management of prostate cancer patients in the year after acquisition.

Private equity's involvement in healthcare has increasingly attracted interest due to uncertainty of how these firm's business model may affect patient care. Some believe that private equity may improve clinical care by reducing administrative responsibilities of physicians, expanding new services available to patients, investing in technology and infrastructure to support care coordination.^10^, ^25^ For example, acquired hospitals perform better with respect to some inpatient quality measures and have lower short‐term mortality compared to nonacquired controls.^26^, ^27^ Nonetheless, prior work in other fields has raised concerns. For example, nursing homes acquired by private equity had higher rates of mortality, a decline in measures of patient well‐being, and increased spending. These outcomes have been attributed to changes implemented by private equity, including staffing reductions among frontline caregivers.^12^ In a different context, acquired dermatology practices increasingly deploy non‐physicians to perform dermatologic exams and procedures, potentially with less accuracy.^28^ Additionally, acquired practices more often perform procedures in patients near the end of life that may have limited clinical benefit.^14^, ^28^ Finally, in dermatology, gastroenterology, and ophthalmology, acquired practices increase productivity by increasing episode costs or throughput (e.g., number of patient visits).^29^ In our national study, private equity acquisition did not lead to an increase in price standardized prostate cancer spending, a global measure of utilization, or treatment. Although spending in the acquired group was significantly higher at baseline ($18,660 vs. $17,145, *p* = 0.045), the acquisition did not affect spending in the year after. Further, in our sensitivity analysis that assessed a nonacquired group consisting of urologists whose practices were eventually acquired demonstrated similar prostate cancer spending per patient at baseline. The subsequent acquisition did not significantly affect spending in the acquired group in this analysis. Though quality was not broadly assessed, treatment in men least likely to benefit did not increase as a result of acquisition.

Private equity is increasingly affecting disciplines that manage patients with cancer, such as medical oncology, radiation oncology, and urology.^6^, ^30^, ^31^, ^32^ These acquisitions could have significant implications at both the practice and market levels, potentially affecting how care is delivered to patients. However, they are generally not reviewed by government agencies such as the Federal Trade Commission. These agencies have limited resources and can only assess the acquisitions they are made aware of, most of which do not meet the required value threshold ($114.4 million in 2023) to disclose.^33^, ^34^ As a consequence, the effect of these acquisitions on local markets may not be realized until years after the transaction, which would make it difficult for patients to make informed decisions about their care. More research is needed to understand the short‐ and long‐term implications of these acquisitions on patient care, physician practice patterns, and healthcare costs. It is also unclear how patients, physicians, and practices fare after the acquired practices are sold to another private equity firm. Until more is understood regarding the effect of these acquisitions, it is important for practices to be transparent with patients on their involvement with for‐profit investors, such as private equity, so patients can make informed decisions about their healthcare.

This study must be interpreted within the context of certain limitations. First, this study only included Medicare beneficiaries, which limits the generalizability of the findings. However, this study includes the majority of men with newly diagnosed prostate cancer based on incidence patterns^35^ and thus reflects the population most affected by changes in behavior due to private equity acquisition. Second, the number of acquired urologists was small and included 11 practices, which likely reduced our statistical power, such that we were unable to detect statistically significant differences that were small or modest between the groups. Nonetheless, the number of patients assessed within these practices was large and we expect to be able to identify major differences in outcomes if they exist. However, as more data become available about the remaining acquisitions (23 out of the 34 as of 2023), future studies should continue to assess these outcomes. This will help determine whether meaningful differences are evident in outcomes that appeared to vary between the groups in this study (e.g., spending). Third, the 12‐month time horizon to assess the effects of acquisition may not have been adequate. Prior studies have assessed changes attributed to private equity beyond the 12‐month period after acquisition and have even excluded the immediate year before and after acquisition as a washout period to avoid capturing the time of acquisition in the analysis.^26^, ^27^ However, due to the recent nature of most acquisitions in urology and the limited availability of Medicare data, it is not currently feasible to assess outcomes beyond 1 year after acquisition in a meaningful way. Additionally, we did not have transaction details for each acquisition, as these are often confidential. Thus, we are unable to account for any differences that may be related to the specific details of the transaction. Despite these limitations, this study demonstrates that private equity acquisition has little immediate effect on the management of prostate cancer.

## 5 CONCLUSIONS

Private equity acquisition of urology practices does not lead to an increase in spending, treatment or use of diagnostic testing for prostate cancer in the year after acquisition. Nonetheless, it is possible that the strategies used by private equity to increase a practice's value may affect treatment patterns beyond the year after acquisition. Thus, with growing involvement of private equity, future work should continue to assess how these acquisitions may affect patient care over a longer time horizon.

## AUTHOR CONTRIBUTIONS

**Kassem S. Faraj:** Conceptualization (equal); formal analysis (equal); methodology (equal); writing – original draft (lead); writing – review and editing (lead). **Samuel R. Kaufman:** Data curation (equal); formal analysis (equal); methodology (equal); resources (equal); software (equal); supervision (equal); validation (equal). **Lindsey A. Herrel:** Conceptualization (equal); investigation (equal); visualization (equal); writing – review and editing (supporting). **Avinash Maganty:** Formal analysis (supporting); investigation (supporting); supervision (supporting); writing – review and editing (supporting). **Mary Oerline:** Data curation (equal); formal analysis (equal); methodology (equal); writing – review and editing (supporting). **Megan E. V. Caram:** Investigation (supporting); supervision (supporting); validation (equal); writing – review and editing (supporting). **Vahakn B. Shahinian:** Conceptualization (equal); funding acquisition (equal); methodology (equal); project administration (equal); supervision (equal); writing – review and editing (equal). **Brent K. Hollenbeck:** Conceptualization (equal); funding acquisition (equal); project administration (equal); supervision (equal); validation (equal); visualization (equal); writing – review and editing (equal).

## FUNDING INFORMATION

This study was supported by funding from NCI T32CA180984 (KF, AM), NCI R01 CA269367 (VS) and a Research Scholar Grant from the American Cancer Society (RSGI‐21‐097‐01‐HOPS).

## CONFLICT OF INTEREST STATEMENT

The authors declare to relevant conflict of interests to the submitted work.

## Supporting information


Figure S1.

Figure S2.

## Data Availability

The data used in this study were obtained from Medicare, a healthcare program in the United States. Access to this data was granted through a data use agreement with the Centers for Medicare Medicaid Services. Due to privacy regulations, the data used in this study are not publicly available. The authors do not have plans for data sharing, due to confidentiality concerns.
