# Supplementary figures and images for: The immediate effects of private equity acquisition of urology practices on the management of newly diagnosed prostate cancer
Source: Cancer Med. 2023 Dec 15;12(24):22325–32. doi: 10.1002/cam4.6788 (PMC10757152; doi:10.1002/cam4.6788)

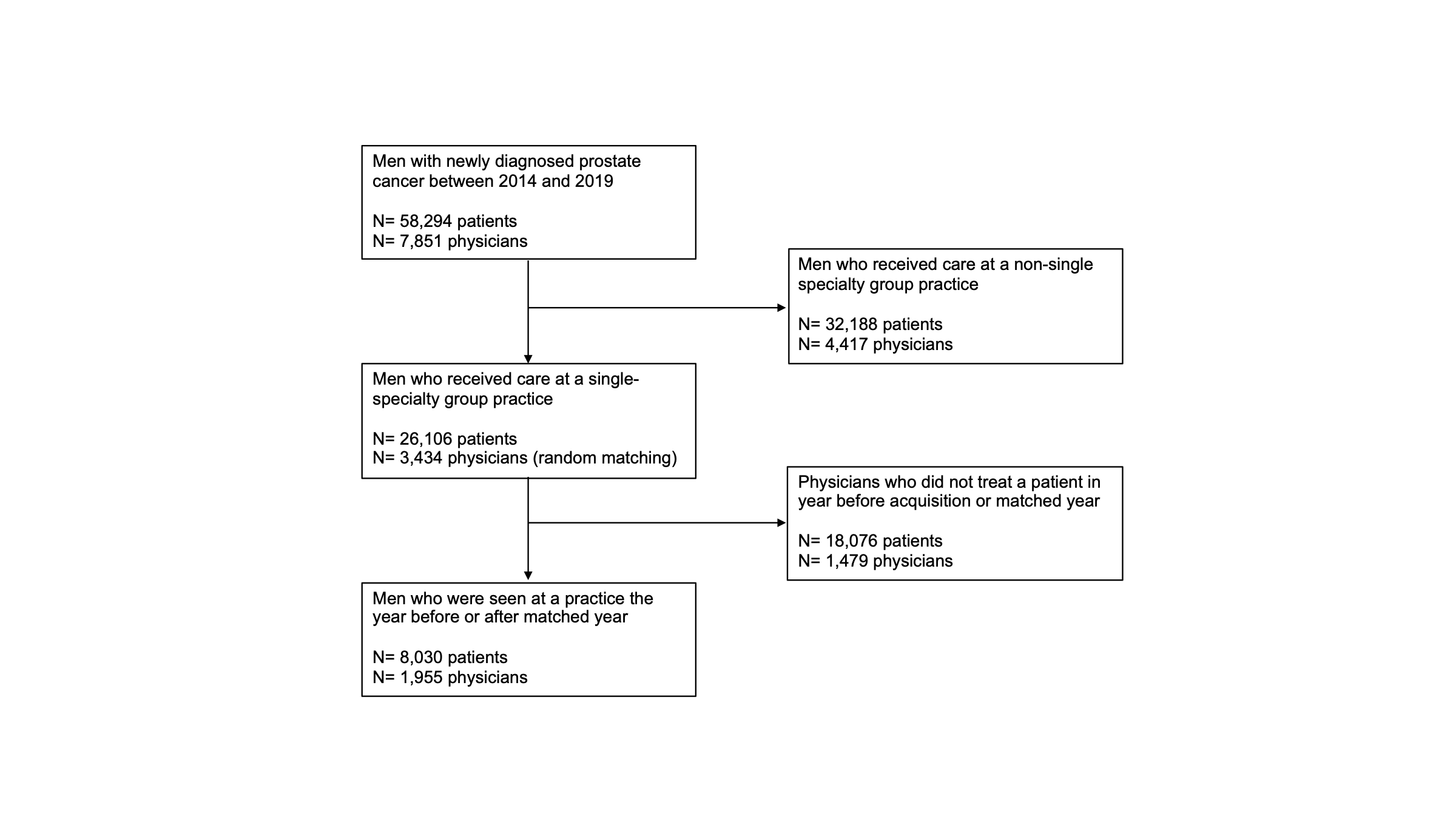

Supplement: Supplementary file 1 — Figure S1. [file CAM4-12-22325-s001.tiff]

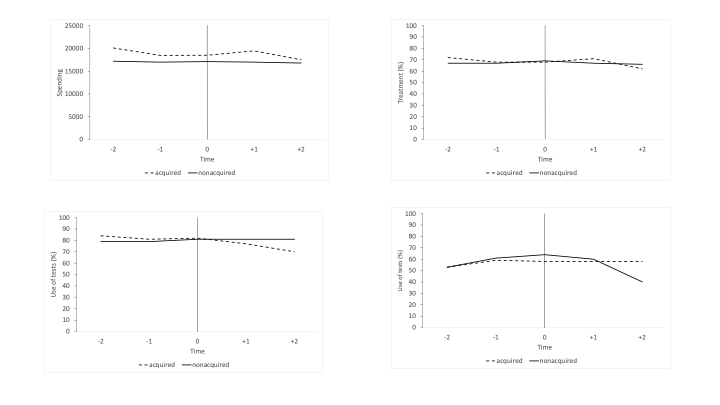

Supplement: Supplementary file 2 — Figure S2. [file CAM4-12-22325-s002.tiff]
